# Tonsil Mycobiome in PFAPA (Periodic Fever, Aphthous Stomatitis, Pharyngitis, Adenitis) Syndrome: A Case-Control Study

**DOI:** 10.3389/fcimb.2020.616814

**Published:** 2021-01-27

**Authors:** Mysore V. Tejesvi, Terhi Tapiainen, Petri Vänni, Matti Uhari, Marko Suokas, Ulla Lantto, Petri Koivunen, Marjo Renko

**Affiliations:** ^1^ Ecology and Genetics, Faculty of Science, University of Oulu, Oulu, Finland; ^2^ Biocenter Oulu, University of Oulu, Oulu, Finland; ^3^ Genobiomics LLC, Oulu, Finland; ^4^ PEDEGO Research Unit, University of Oulu, Oulu, Finland; ^5^ Department of Paediatrics and Adolescent Medicine, Oulu University Hospital, Oulu, Finland; ^6^ Department of Otorhinolaryngology, Oulu University Hospital, Oulu, Finland; ^7^ Department of Paediatrics, University of Eastern Finland and Kuopio University Hospital, Kuopio, Finland

**Keywords:** mycobiome, tonsil, PFAPA, machine learining, next generation sequencing

## Abstract

Periodic fever, aphthous stomatitis, pharyngitis and adenitis syndrome (PFAPA) is the most common periodic fever syndrome in children with unknown etiology, effectively treated with tonsillectomy. Earlier we have shown that tonsil microbiome is different in patients with PFAPA as compared to that in controls. Recently, fungal microbiome, mycobiome, has been linked to the pathogenesis of inflammatory diseases. We now investigated the role of mycobiome of tonsils in PFAPA. Random forest classification, a machine learning approach, was used for the analysis of mycobiome data. We examined tonsils from 30 children with PFAPA and 22 control children undergoing tonsillectomy for non-infectious reasons. We identified 103 amplicon sequence variants, mainly from two fungal phyla, Ascomycota and Basidiomycota. The mean relative abundance of *Candida albicans* in the tonsil mycobiome was 11% (95% CI: 19 to 27%) in cases and 3.4 % (95% CI: -0.8% to 8%) in controls, p =0.104. Mycobiome data showed no statistical difference in differentiating between PFAPA cases and controls compared to a random chance classifier (area under the curve (AUC) = 0.47, SD = 0.05, p = 0.809). In conclusion, in this controlled study, tonsillar mycobiome in children with PFAPA syndrome did not differ from that of the controls.

## Introduction

Periodic fever, aphthous stomatitis, pharyngitis and adenitis (PFAPA) is a childhood febrile syndrome of unknown origin in which fever flares occur in regular 3- to 5-week cycles. Between febrile episodes, patients are asymptomatic (Marshall et al., 1989; Thomas et al., 1999). Although PFAPA syndrome has been suggested to be an autoinflammatory disorder due to dysregulated cytokine production in inflammasomes (Brown et al., 2011; Stojanov et al., 2011; Kolly et al., 2013), its etiology remains unknown. PFAPA is likely a polygenic or complex genetic disease and more recognized in adult patients (Cantarini et al., 2012; Adrovic et al., 2019; Manthiram et al., 2020). Even though randomized controlled studies have shown that tonsillectomy (TE) is a curative treatment for PFAPA syndrome (Renko et al., 2007; Garavello et al., 2011), the mechanism of this effect remains unclear. Earlier we have shown that tonsil microbiome is different in patients with PFAPA as compared to that in controls (Tejesvi et al., 2016).

In healthy hosts, the host and commensal microbiomes are characterized by interaction and homeostasis (Levy et al., 2015). Earlier, microbiome research has mostly focused on the impact of the bacterial microbiome, referred to as bacteriome, on health (Iliev et al., 2012; Tejesvi et al., 2016; Sokol et al., 2017). However, recent research has drawn attention to the importance of host-associated fungi, the mycobiome, in the inflammatory processes of the human body (Ward et al., 2017). Changes in the mycobiome have been associated with the modulation of autoinflammatory immune responses and disease progression, for example, in Crohn’s disease (El Mouzan et al., 2018). Furthermore, the mycobiome may be involved in the host immune response and constitute a risk factor for immunological disorders (Kumamoto, 2016). Finally, it may function as a reservoir of opportunistic pathogens, such as *Candida albicans*, in immunocompromised patients (Polvi et al., 2015; Huseyin et al., 2017).

The human gut mycobiome has been associated with chronic inflammatory diseases of the gut, with studies focusing on intestinal fungi (Iliev et al., 2012; Sokol et al., 2017). However, data on oral mycobiome is scarce. In this controlled study, our main objective was to investigate the role of the mycobiome in tonsils as a potential trigger of inflammatory responses in PFAPA syndrome using next-generation fungal microbiome sequencing technology. Furthermore, random forest classification, a machine learning approach, was used for mycobiome data in classifying PFAPA cases and controls.

## Materials and Methods

### Recruitment of the Patients and Controls

Between March 2006 and April 2010, we recruited 30 consecutive patients (median age: 3.4 years; **Table 1**) who underwent TE due to PFAPA. The diagnostic criteria; i.e. at least five episodes of high fever of unknown origin recurring with a typical, regular pattern and asymptomatic intervals of 2 to 5 weeks, were the same as in our previous randomized controlled study on TE in PFAPA (Renko et al., 2007). During the same period, 22 children (median age: 5.6 years) undergoing TE due to hypertrophied tonsils were recruited as controls.

**Table 1:** Demographic characteristics of pharyngitis and adenitis syndrome (PFAPA) cases and controls.

	PFAPA (N = 30)	Controls (N = 22)
Age when symptoms began, median (range), years	2.3 (0.1–16.5)	
Age at the time of surgery, median (range), years	3.4 (1.7–18.2)	5.6 (2.7–15.2)
Gender, boys, N (%)	18 (60%)	8 (36%)
Antimicrobial courses mean (SD)		
Within 12 months prior to TE	2.5 (2.6)	1.3 (1.6)

Data on age at onset of symptoms, time of surgery, and antimicrobial course are presented.

### Demographics of the Patients and Controls

Data on the patients’ symptoms were collected before surgery using a questionnaire. The median age of the PFAPA patients at the onset of the fever periods was 2.3 years. The average duration of PFAPA symptoms before TE was 12 months. The mean maximum fever was 39.7°C, and the mean duration of the febrile episodes was 3.9 days. The mean time interval between two subsequent febrile episodes (start to start) was 26 days. None of the patients received steroids for PFAPA prior to TE: The median age at the time of surgery was 3.4 years in the PFAPA group and 5.6 years in the control group. In all PFAPA patients, the symptoms were resolved after TE.

We obtained data on the children’s use of antimicrobials in the 12 months before tonsillectomy from the Finnish National Drug Purchase Register, maintained by the Social Insurance Institution of Finland (Kela). Exposure to antimicrobials in the 12 months before tonsillectomy was greater in the PFAPA group (mean number of antimicrobial courses: 2.5) than in the control group (1.3). However, the difference was not statistically significant (95% confidence interval: −0.1, 2.4; p = 0.07; **Table 2**).

**Table 2:** The mean relative abundances of most abundant fungal species in pharyngitis and adenitis syndrome (PFAPA) children and controls.

Fungi	PFAPA (%)	Control (%)	p-value*^a^*
	(N = 30)	(N = 22)	
*Malassezia* spp	11.38	14.37	0.789
*Candida_albicans*	10.90	3.43	0.190
*Malassezia_restricta*	9.78	4.16	0.411
Helotiales	6.08	1.66	0.319
*Rhexocercosporidium_panacis*	3.24	4.43	0.768
Herpotrichiellaceae	2.82	3.20	0.122
*Malassezia_globosa*	2.07	6.67	0.792
*Tomentella_sublilacina*	1.69	5.12	0.105
*Cladosporium*	1.03	1.79	0.871
*Suillus_bovinus*	0.09	3.10	0.347
Dermateaceae	0.04	4.15	0.076
*Masticobasidum*	0.00	0.90	0.039

The mean relative abundances are shown as percentages.

a p-values calculated using Mann-Whitney U test.

The parents of all patients provided written informed consent. This study’s protocol was approved by the Ethics Committee of the Northern Ostrobothnia Hospital District, Oulu, Finland. All methods were carried out following relevant guidelines and regulations.

### The Samples and DNA Extraction

A total of 30 tonsil samples from PFAPA patients and 22 control samples were stored at −80°C and later used for mycobiome and machine learning analyses. The microbiology of these samples, as well as the details of the patients and the operations, have been described previously (Lantto et al., 2015; Tejesvi et al., 2016). All microbiological analyses were performed blinded to indications for TE.

For the mycobiome analyses, we isolated DNA from the 54 tonsil samples, from 30 cases and 24 controls. DNA was extracted from the tonsil samples using a Quick-DNA Fungal/Bacterial Miniprep Kit (Zymo Research, USA) according to the manufacturer’s protocol. The quantity and quality of DNA were determined using a NanoDrop 1000 spectrophotometer (Thermo Scientific, Waltham, MA, USA).

### Amplification of the Fungal Internal Transcribed Region

The fungal internal transcribed spacer 2 (ITS2) region was amplified using ITS7b and ITS4 primers including an Ion Torrent pyrosequencing adapter with a 10-bp barcode sequence to the ITS4 primer. Polymerase chain reactions (PCR) were performed in three replicates, each containing a 1x Phusion HF buffer, 0.4 µM of forward and reverse primers, 200 µM of dNTPs, 0.5 U of Phusion enzyme (Thermo Scientific, Finland) and 20 ng of genomic community DNA as the template and molecular-grade water in a total reaction volume of 20 µl. After an initial denaturation at 98°C for 3 min, the following cycling conditions were used: 38 cycles of 98°C, 10 s; 56°C, 10 s; and 72°C, 20 s. After PCR amplification, the pooled triplicate reactions were purified using an AMPure XP PCR cleanup kit (Agencourt Bioscience, CA, USA) and assessed for DNA size, molarity and quality using an Agilent Bioanalyzer 2100 (Agilent Technologies, CA, USA). Finally, the samples were diluted to equimolar concentrations and sequenced using an Ion 316 Chip Kit v2 with the Ion Torrent PGM platform (Thermo Fisher, Life Technologies, USA).

### Mycobiome Analysis

Denoising and amplicon sequence variants (ASV) picking were performed using the default settings of the DADA2 algorithm in QIIME2 (Callahan et al., 2016). Chimeric sequences were removed from the data with the q2-vsearch plugin implementing VSEARCH in QIIME2. Sequences in the data that were not classified into the kingdoms of fungi were removed using a custom Python script. ASVs found only in one sample and had lower than 100 reads across the table were excluded using a feature-table plugin in QIIME2. Two samples had a low number of reads and were excluded from further analysis. Taxonomic classification of fungal sequences was performed using Naive Bayes classifiers with the q2-feature-classifier plugin, trained on an ITS silva database for fungi. Phylogenetic trees were created using the q2-phylogeny plugin, which utilizes MAFFT and FastTree (Katoh and Standley, 2013). Alpha and beta diversity analyses were performed using a rarefying depth of 650 for fungi with the q2-diversity plugin and visualized with custom scripts using the Matplotlib package for Python (Hunter, 2007). Statistical comparisons between groups were performed with custom scripts using the SciPy package for Python (Oliphant, 2007). The observed ASVs, Pielou’s evenness, Shannon’s diversity index and Faith’s phylogenetic diversity were chosen as alpha diversity metrics. Principal coordinate analyses were performed for Bray-Curtis, Jaccard and weighted and unweighted UniFrac distances and visualized in two dimensions. A Venn diagram was drawn using an online tool at Euler Venn Applet using differential abundance data between controls and PFAFA cases (http://bioinformatics.psb.ugent.be/webtools/Venn/). Pie charts of fungal taxonomy were created using Krona (Ondov et al., 2011).

### Machine Learning Analysis

Random forest (Breiman, 2001) classifiers were trained on relative abundance tables collapsed to the genus level to differentiate between control and PFAFA case samples based on fungal data. The classifiers’ performance was assessed with out-of-bag accuracy and receiver operating characteristic (ROC) curves averaged over stratified tenfold cross-validation on the whole dataset. For the averaged ROC curve, 95% confidence intervals were calculated using a Bayesian method in the Scipy package (Oliphant, 2007). The feature importance of different ASVs was assessed using mean decrease impurity (MDI), where Gini is the impurity metric, averaged for each cross-validation. The classifiers were used to predict the same test set with real and shuffled labels in each fold. The averaged area under the curve (AUC) from each cross-validation for real and shuffled labels was tested with an independent t-test. The entire process was repeated 100 times, the values were averaged, and the resulting 100 p-values were combined using Fisher’s method. Samples that had zero relative abundance for every variable were excluded. The machine learning analysis was performed with custom Python scripts using the scikit-learn package (Pedregosa et al., 2011), and figures were plotted with Matplotlib 3.2.1 (Hunter, 2007).

### Statistical Analyses

For sample size calculation we anticipated to find out a 40% difference in the presence of *C. albicans* in the tonsils between the groups. With an alfa error of 5% and power of 80%, we calculated a sample size of 24 patients per group to be needed.

We used Student’s t-test for independent samples to compare the mean number of observed ASVs and the means of the indices describing the diversity of the mycobiome between PFAFA patients and controls. We calculated the means (or medians) with their standard deviations (SDs) (or ranges) of the relative abundances of fungal phyla, the most abundant genera and selected genera in each group. The statistical significances of the differences were tested using the nonparametric method with the Mann-Whitney U test. The analyses were performed using IBM SPSS Statistics 25 (IBM, Armonk, NY, USA).

## Results

### General Description of the Mycobiome in Tonsil Samples

**Figure 1:**
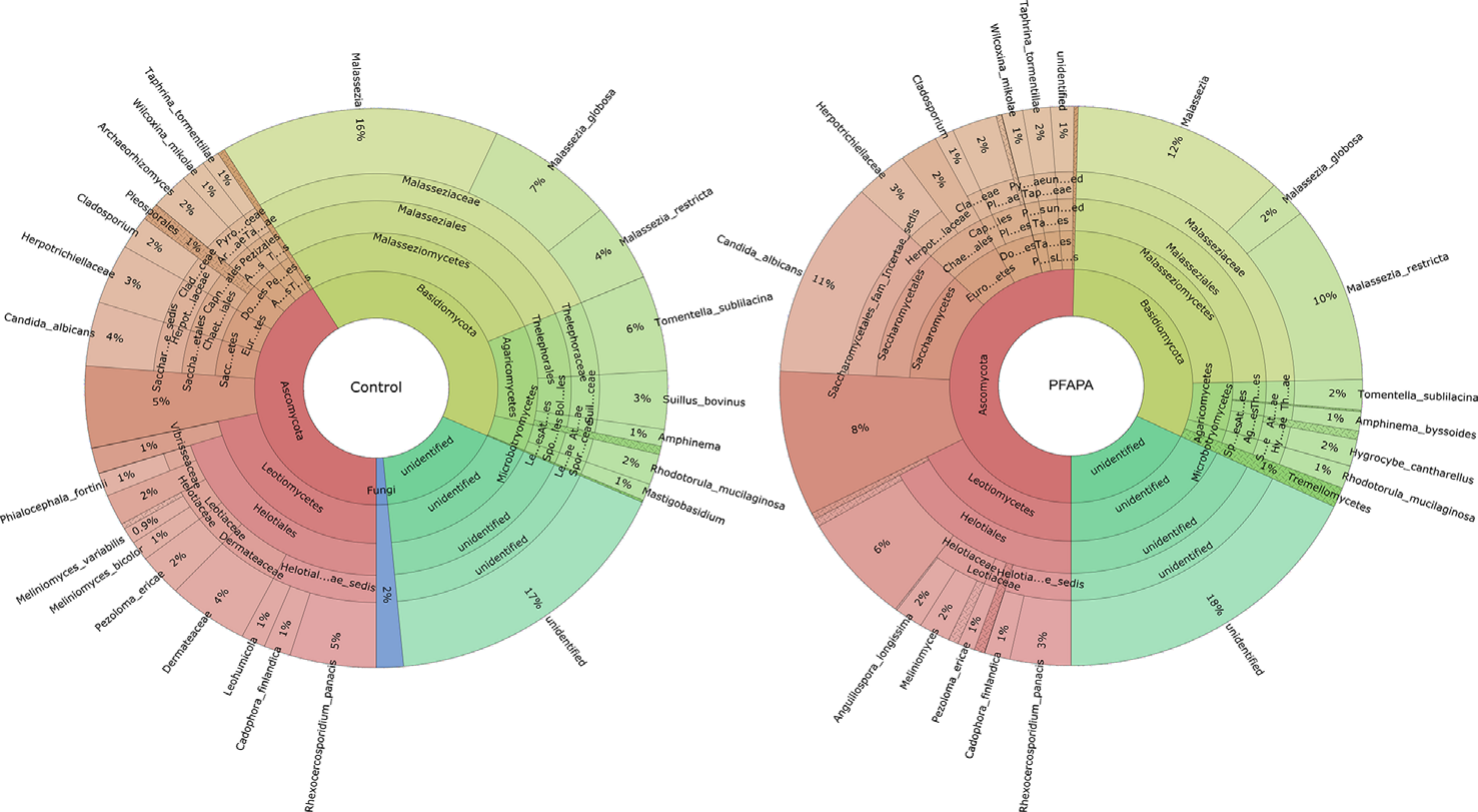
The relative abundance of fungal mycobiome community in 52 tonsil tissue samples [30 pharyngitis and adenitis syndrome (PFAPA) and 22 controls]. Less abundant (<0.5 %) genera are not shown or are combined.

Across all samples, we identified 103 amplicon sequence variants (ASVs) from two major fungal phyla, Ascomycota and Basidiomycota, and some unclassified reads. Sequence reads belonging to the phyla Basidiomycota and Ascomycota were present in all samples. The number of ASVs per sample ranged from 2 to 18. The most abundant genera belonging to the phylum Ascomycota were *Candida*, *Gyoerffyella*, *Meliniomyces* and *Rhexocercosporidium*, while those belonging to the phylum Basidiomycota were *Malassezia*, *Telephora*, *Suillus* and *Rhodotorula* (**Figure 1** and **Supplementary Figure 1**). The relative abundance of representative genera, class and family of control and cases are presented in **Table 2**.

## Comparisons Between the Tonsil Mycobiomes of Periodic Fever, Aphthous Stomatitis, Pharyngitis, and Adenitis Syndrome Cases and Controls

**Figure 2:**
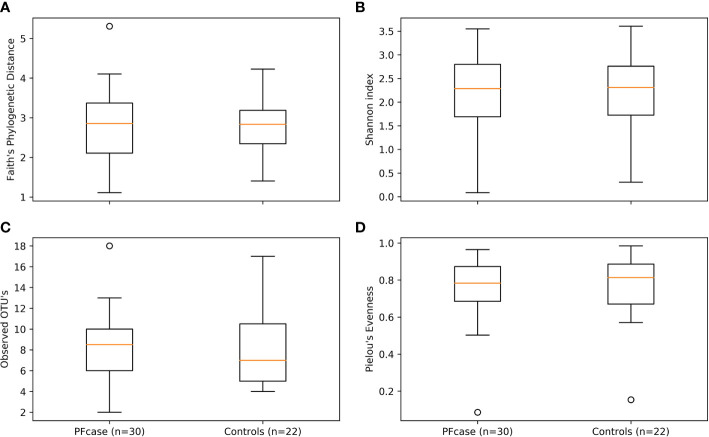
Alpha diversity boxplots of PFcase and control samples. Metrics used are **(A)** Faith’s phylogenetic distance, **(B)** Shannon index, **(C)** Observed amplicon sequence variant’s (ASV’s) and **(D)** Pielou’s evenness. Outlier samples are shown as dots.

**Figure 3:**
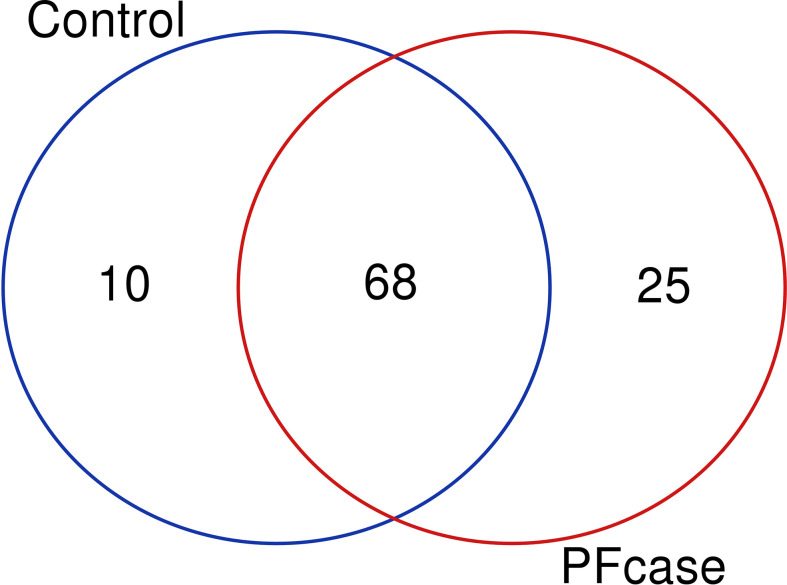
Venn diagram illustrating overlap and number of unique taxa between controls and PFcases. The red and green circles represent PFcases and controls respectively.

**Figure 4:**
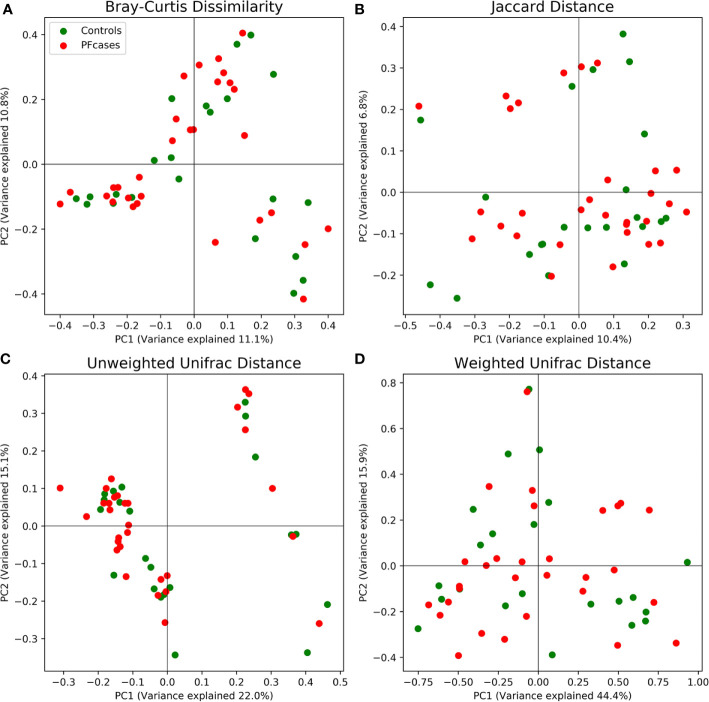
Principal coordinate analysis results plotted for two of the most variance explaining components. Red and green dots represent PFcases and controls, respectively. In **(A)** Bray-Curtis dissimilarity, **(B)** Jaccard distance, **(C)** unweighted Unifrac distance and **(D)** weighted Unifrac distance are shown.

There were no significant differences in the number of ASVs or the alpha diversity indices between tonsil samples from PFAPA cases and controls (**Figure 2**). Both groups shared 68 unique taxa, while 25 ASVs were present only in the cases and 10 only in the controls (**Figure 3**). The most abundant phyla in both groups were Basidiomycota and Ascomycota. Basidiomycota were more prevalent in cases and Ascomycota in controls; however, the differences were not statistically significant (**Figure 1**). The Bray-Curtis dissimilarity, Jaccard distance and weighted and unweighted UniFrac distances showed slight clustering in the top two respective principal coordinates. However, they did not differentiate between PFAPA and controls (**Figure 4**).

### Microbial Diversity and Comparison of *Candida albicans* Abundance

At the species level, *C. albicans* was present in 43% of the cases and 27% of the controls (p = 0.235). The mean relative abundance of *Candida albicans* was 11% (95% CI: 19 to 27%) in cases and 3.4 % (95% CI: -0.8% to 8%) in controls, p =0.104. There were no statistically significant differences in the mean abundance of other fungal species between the cases and controls. The indices describing alpha diversity and the relative abundance of the phyla did not differ significantly in subjects with or without antibiotic courses during the year before tonsillectomy.

### Machine Learning Analysis of the Mycobiome

**Figure 5:**
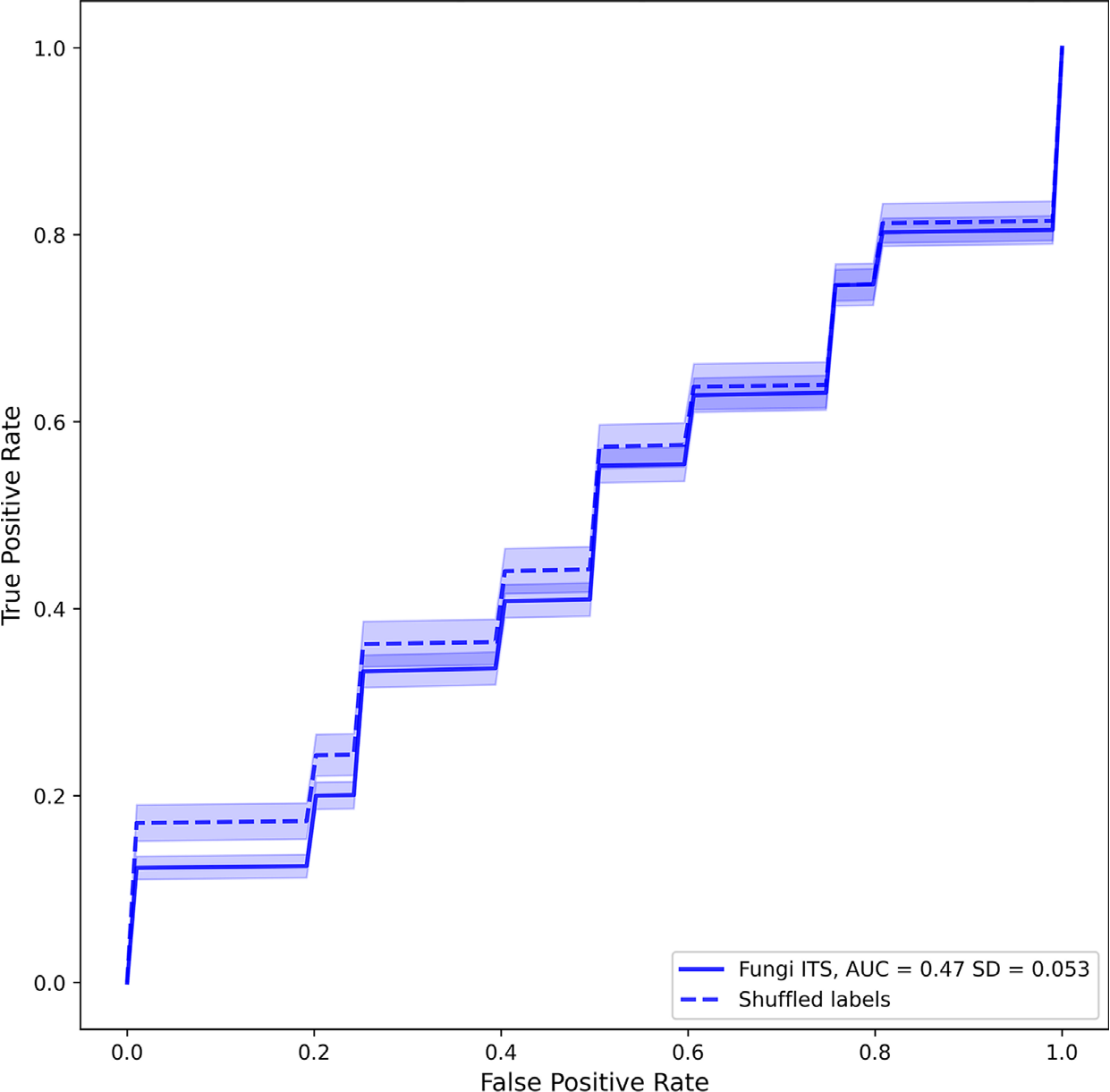
Averaged receiver operating characteristic curves of fungal classifiers trained to differentiate positives (PFcase samples) from negatives (control samples). Solid and dashed lines represent real and shuffled labels. Transparent areas around each line represent the 95% confidence intervals of the curve.

A classifier was trained to differentiate cases from controls according to the mycobiome data collapsed to the genus level using the silva database. The random classifier Random forest differentiation between PFAPA cases and controls showed no statistical difference in performance compared to a random chance classifier (area under the curve (AUC) = 0.47, SD = 0.05; p = 0.809; **Figure 5**).

## Discussion

We have earlier shown that tonsil bacterial microbiome composition is associated with PFAPA syndrome (Tejesvi et al., 2016). In the previous study, only bacterial microbiome was investigated. We hypothesized that mycobiome may associate with the pathogenesis of PFAPA as well. In this controlled study, we showed tonsil mycobiome and its composition in PFAPA and controls. However, we could not confirm the association of mycobiome with the pathogenesis of PFAPA syndrome. Machine learning analysis performed on mycobiome data did not classify PFAPA cases and controls.

PFAPA has been suggested to be an autoinflammatory disease, a condition characterized by abnormally high or uncontrolled inflammation (Wekell et al., 2016). *Candida albicans* is one of the strongest and best verified triggers of inflammasome activity (Joly et al., 2009; Rehaume et al., 2010). It can stimulate inflammasomes, especially in hyphenal forms (Joly et al., 2009). It has also been suggested to play a role in the pathogenesis of inflammatory gut diseases (Sokol et al., 2017). Using culture-based methods and electron microscopy, we previously reported more culture-positive *C. albicans* findings and biofilm formation in tonsillar tissue of PFAPA patients than in tissue of controls (Lantto et al., 2015). In another case-control study, we found that PFAPA patients report clinical oral thrush, an oral fungal infection, in their medical histories more frequently than healthy controls (Lantto et al., 2018). In this study, we hypothesized that the excessive presence of *C. albicans* observed in our previous culture-based study would be even more evident with modern sequencing and machine learning techniques (Rehaume et al., 2010; Wekell et al., 2016; Kocheturov et al., 2019). *Candida albicans* was indeed more abundant in the tonsil mycobiome of PFAPA patients than of controls, but the difference was not statistically significant. Thus, we could not confirm the hypothesis with our sample size; however, the observed difference warrants further studies. Notably, a fluctuation in the inflammatory response might result in a fluctuation in the abundance of microbial triggers as well.

Using traditional statistical models, it is difficult to identify the fungal populations associated with a disease. There is moreover significant variation in microbiome structures between individuals (Walters et al., 2014). To overcome these challenges, machine learning analysis is now being widely used (Johnson et al., 2016; Ai et al., 2017). In our earlier studies on the pathogenesis of PFAPA, we used conventional microbiology of tonsils (Lantto et al., 2015) and then microbiome analysis with conventional statistics (Tejesvi et al., 2016). In this study, we used a machine learning approach and our results does not show a significant difference in discerning PFAPA cases between machine learning analysis and random chance.

Before the next-generation sequencing era, mycobiome was traditionally investigated by *in vitro* culturing. Significant discrepancies in culture-dependent and independent methods are likely, as most fungi are not cultivable in laboratory media (Chen et al., 2011; Browne et al., 2016). Recent studies using next-generation sequencing technology have overcome the bias of culture-dependent methods but have mostly focused on the bacteriome. Recent studies have investigated the role of the human mycobiome in the pathogenesis of gastrointestinal diseases in immunocompromised hosts, diabetes and obesity, revealing the functional diversity of fungi associated with different human body sites (Mukherjee et al., 2014; Kowalewska et al., 2016).

The mycobiome may vary in different parts of the tonsillar tissue, namely, in the superficial layers and the crypts. However, we were unable to determine whether samples for DNA extraction had been taken from superficial layers or crypts.

Fungal dysbiosis and homeostasis are dynamic processes that are probably more common than actual fungal infections and therefore continually shape the immune response (Iliev and Leonardi, 2017). Diversity in the oral mycobiota is lower than in the bacteriome. In healthy adults, the oral mycobiota is dominated by members of the Ascomycota phylum, mainly *Candida* species (Ghannoum et al., 2010; Bandara et al., 2019). These phyla are also dominant in other parts of the human body (Nash et al., 2017). In a culture-based study, about 12% of infants were found to be oral carriers of *Candida* species (Stecksen-Blicks et al., 2015). However, the oral mycobiome of small children has not been studied with modern techniques. *Candida albicans* is more prevalent in oral samples of patients with rheumatoid arthritis (Bishu et al., 2014) and periodontal disease than in control samples (Peters et al., 2017).

Th17 cells are known to participate in host defense against fungi and extracellular bacteria, and their role in maintaining homeostasis between commensal microorganisms and the host has also been studied (Zielinski, 2014). In a study on the differentiation of Th17 cells *in vivo*, *C. albicans* induced pro-inflammatory Th17 cells that produced IL-1b. However, these cells were incapable of self-regulatory IL-10 production. In our previous culture-based study, tonsil samples from PFAPA patients yielded *C. albicans* more often than those from controls. These results may indicate an important role of the tonsil microbiota in the pathogenesis of PFAPA syndrome.

The role of the mycobiome in various diseases is still largely unknown. This was the first study to examine the mycobiome of children’s tonsils. The strength of this study was its controlled design. One limitation was that it was not possible to collect control samples from healthy children. Thus, we chose to use children with hypertrophic tonsils as controls. Another limitation was that the mean age of the control group was older than that of the patient group. Moreover, the sample size was not large enough to perform analyses stratified by age. Furthermore, exposure to different antimicrobials before tonsillectomy may have affected our microbiological findings. In our series, the cases had more often received antimicrobial courses 12 months before tonsillectomy; however, the difference was not statistically significant.

In conclusion, the tonsil mycobiome of PFAPA children did not statistically differ from that of controls in this study. Thus, we could not confirm that *Candida*, earlier associated with PFAPA in epidemiological and conventional microbiological studies, is a trigger of excessive, fluctuating inflammatory response to the mycobiome in the tonsils in PFAPA. The tonsil mycobiome is less diverse and candida is the major genera present in the tonsils. Machine learning performed on mycobiome data did not classify PFAPA cases. However, future studies with a larger sample size may classify and accurately predict PFAPA cases.

## Data Availability Statement

We have deposited the Ion Torrent fungal raw data in the Sequence Read Archive (SRA) with accession number SRP132771.

## Ethics Statement

The studies involving human participants were reviewed and approved by the Ethics Committee of the Northern Ostrobothnia Hospital District, Oulu, Finland. Written informed consent to participate in this study was provided by the participants' legal guardian/next of kin.

## Author Contributions

MR, TT, UL, and PK planned the clinical study and tonsil sample collection. MT, TT, PV, and MR wrote the manuscript. MT did the laboratory work, and MS sequenced the mycobiome. MT and PV performed the bioinformatics analysis. MU critically reviewed the manuscript. All authors contributed to the article and approved the submitted version.

## Funding

This work was supported by the Society for Pediatric Research and the Finnish Medical Foundation.

## Conflict of Interest

Authors MT and PV are co-founders of the company Genobiomics LLC, Finland.

The remaining authors declare that the research was conducted in the absence of any commercial or financial relationships that could be construed as a potential conflict of interest.

## Supplementary Material

Supplementary Figure 1 The relative abundance of most abundant taxa in the control and PFcase.
