# Supplementary figures and images for: Tonsil Mycobiome in PFAPA (Periodic Fever, Aphthous Stomatitis, Pharyngitis, Adenitis) Syndrome: A Case-Control Study
Source: Front Cell Infect Microbiol. 2021 Jan 27;10:616814. doi: 10.3389/fcimb.2020.616814 (PMC7873641; doi:10.3389/fcimb.2020.616814)

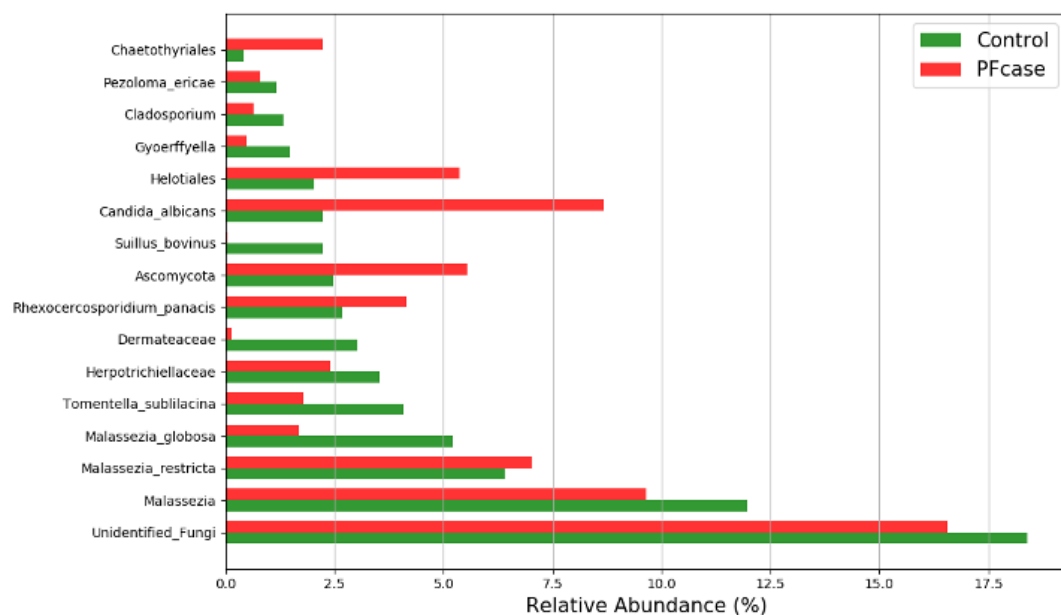

**Supplementary Figure 1. The relative abundance of most abundant taxa in the control and PFcase.**

Supplement: Supplementary Figure 1 — The relative abundance of most abundant taxa in the control and PFcase. [file DataSheet_1.pdf]
